# Using Mendelian randomization analysis to determine the causal connection between unpleasant emotions and coronary atherosclerosis

**DOI:** 10.3389/fcvm.2023.1126157

**Published:** 2023-05-22

**Authors:** Jiyong Lei, Da Luo, Jiarui Xiong, Mingjiang Li

**Affiliations:** ^1^ Department of Cardiology, Renmin Hospital of Wuhan University, Wuhan, China; ^2^ Cardiovascular Research Institute, Wuhan University, Wuhan, China; ^3^ Hubei Key Laboratory of Cardiology, Renmin Hospital of Wuhan University, Wuhan, China

**Keywords:** unpleasant emotions, anxiety, depression, coronary atherosclerosis, Mendelian randomization

## Abstract

**Objective:**

Observational studies have shown a correlation between unpleasant emotions and coronary atherosclerosis, but the underlying causal linkages are still uncertain. We conducted a Mendelian randomization (MR) investigation on two samples for this purpose.

**Methods:**

In genome-wide association studies in the UK Biobank (total = 459,561), we selected 40 distinct single-nucleotide polymorphisms (SNPs) related to unpleasant emotions as genome-wide statistically significant instrumental variables. FinnGen consortium provided summary-level data on coronary atherosclerosis for 211,203 individuals of Finnish descent. MR-Egger regression, the inverse variance weighted technique (IVW), and the weighted median method were used in the process of conducting data analysis.

**Results:**

There was sufficient evidence to establish a causal connection between unpleasant emotions and coronary atherosclerosis risk. For each unit increase in the log-odds ratio of unpleasant feelings, the odds ratios were 3.61 (95% CI: 1.64–7.95; *P* = 0.001). The outcomes of sensitivity analyses were comparable. There was no indication of heterogeneity or directional pleiotropy.

**Conclusion:**

Our findings provide causal evidence for the effects of unpleasant emotions on coronary atherosclerosis.

## 1. Introduction

Coronary atherosclerosis, commonly known as coronary artery disease (CAD), is the greatest frequent form of cardiovascular disease (CVD). Typically, the growth of atherosclerotic lesions lowers lumen blood flow by more than 50%, resulting in angina, myocardial infarction (MI), or stroke, particularly during exercise or other stressful conditions (1). Despite its slow development, atherosclerosis continues to be one of the primary causes of death around the globe (1). With more women, younger people, and multiethnic groups being impacted by atherosclerosis than ever before, and with non-traditional risk factors garnering attention, new observational research has improved our knowledge of the disease (2). Evidence from the INTERHEART trial which is a major, globally standardized case-control research evaluating global risk factors for coronary heart disease, encompassing 262 sites from 52 nations reveals that stress and depression are non-negligible CVD risk factors, accounting for 32.5% of attributable risk (3). In the Pelotas Birth Cohort of 1993, there were 5, 249 people and 4, 336 persons with full mental health data, Belem da Silva et al. studied the association between carotid intima-media thickness(cIMT) at age 18 and emotional symptoms (ESs) referring to depression or anxiety symptoms which were assessed by the Strengths and Difficulties Questionnaire (SDQ) (4) at ages 11 and 15 years (5). High expression of ESs in children aged 11 and 15 was associated with 1.84 μm and 2.58 μm, respectively, an increase in cIMT at 18 years of age (*P *< 0.001) (5). It indicates that the intensity of ESs has predictive value for the risk of future atherosclerosis (5). However, contradictory findings were discovered regarding the effect of psychological disorders, such as anxiety and depression, on subclinical atherosclerosis. Using cIMT, Prugger et al. observed a longitudinal association between subclinical atherosclerosis and the progression of depression symptoms in patients who are older during a follow-up period of ten years (6). In contrast, other cross-sectional studies have not discovered this connection (7). Due to the nature of observational studies, the potential for bias in these findings as a consequence of residual confounding, and the ambiguity of causation between data, we undertook a Mendelian randomization study (MR) to investigate the causality between unpleasant emotions, such as symptoms of nerves, anxiety, tension or depression, and coronary atherosclerosis for observational findings cannot be determined.

## 2. Materials and methods

**Figure 1:**
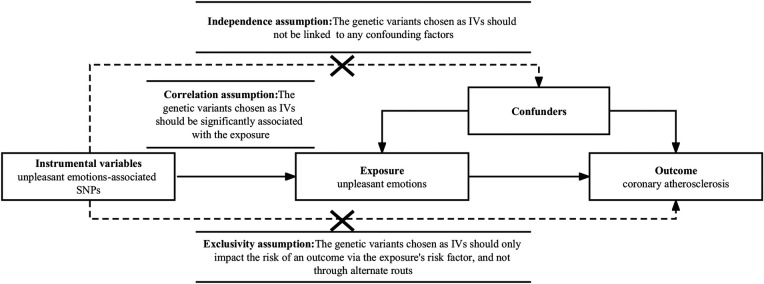
Design framework and assumptions of the Mendelian randomization study. Independence assumption, the genetic variants chosen as instrumental variables(IVs) should not be linked to any confounding factors; correlation assumption, IVs should be significantly associated with the exposure; exclusivity assumption, IVs should only impact the risk of an outcome *via* the exposure's risk factor, and not through alternate routes. SNPs, single nucleotide polymorphisms.

The Mendelian randomization studies (MR) are based on the idea of instrumental variables (IVs) and use Single Nucleotide Polymorphisms (SNPs) to explore the causal relationship between exposure and outcome. Because gamete formation follows the Mendelian law of random assignment of parental alleles to offspring, environmental exposures, socioeconomic level, and behavioral characteristics do not influence genetic diversity; moreover, genetic variation remains constant after birth, providing an estimate of the impact of risk factors over the lifetime of the individual. Therefore, MR can overcome the confounding and reverse causality problems that exist in traditional observational studies (8). MR is predicated on three key premises. Initially, the genetic variations selected as IVs should be strongly related to the exposure. Second, the genetic variations selected as IVs should not be connected to any confounding variables. Lastly, the genetic variants chosen as IVs should only impact the risk of an outcome via the exposure's risk factor, and not through alternate routes (9) (Figure 1).

### 2.1. Standards and definitions

Unpleasant emotions consist of two distinct states: the first is a state of psychological non-relaxation caused by poor interpersonal relationships or other factors, such as fear, anxiety, or anger in the face of challenges and the unknown; the second is a state of low mood, loss of interest or pleasure that does not correspond to the current situation (10).

The process of lipid deposition, complex glycan accumulation, fibrous tissue proliferation, and calcium deposition is known as coronary atherosclerosis (11). In the end, it leads to arterial wall thickening, lumen constriction, and blood flow obstruction, resulting in myocardial ischemia and hypoxia (11).

### 2.2. Data sources

We used second-wave UK Biobank data from the Bristol Medical School's MRC-IEU(Medical Research Council, MRC; Integrative Epidemiology Unit, IEU), which altered the variant call format to hold aggregated GWAS statistics (GWAS-VCF) to enhance the effectiveness of GWAS summary data queries and lower the likelihood of data interpretation and post-GWAS analysis mistakes on genetic information about unpleasant emotions (12, 13). The group consisted of 459,560 persons of European ancestry, of whom 158,565 had ever visited a general practitioner (GP) for the symptoms of nerves, anxiety, tension, or depression. Then, we utilized summary data from the FinnGen R7 version of the GWAS results to examine the relationship between unpleasant emotions and coronary atherosclerosis in up to 211,203 persons of Finnish heritage (14). The 8th and 10th editions of the International Classification of Diseases (ICD) identify coronary atherosclerosis. These data sets were collected from the Bristol Medical School's Open Access Complete GWAS Summary Data Set (https://gwas.mrcieu.ac.uk/) (12). It has been suggested that emotions are associated with coronary atherosclerosis risk factors, but it is unknown whether these risk factors mediate the influence of emotions on coronary atherosclerosis (15–18). We enumerated the potential confounding factors (Table 1). To ensure the efficacy of the MR analysis, the phenoscanner (http://www.phenoscanner.medschl.cam.ac.uk/) was then utilized to examine the possibility of pleiotropy.

**Table 1:** Potential confounding variables for coronary atherosclerosis.

Trait or disease	Study
Sex	Bangasser et al. (17)
Hypercholesterolemia	Blöchl et al. (15)
Hypertension	Blöchl et al. (15)
Smoke	Taylor et al. (18)
Diabetes	Blöchl et al. (15)
Obesity	Chaplin et al. (16)

### 2.3. Selection for genetic variation

Single-nucleotide polymorphisms (SNPs) related to unpleasant emotions at the genome-wide significance level (*P *< 5 × 10^−8^) were retrieved from GWASs of 459,560 participants of European ancestry from UK Biobank (13). Then we utilized 1, 000 genome linkage disequilibrium European groups as reference populations to assess whether these SNPs exhibit linkage disequilibrium (LD r^2 ^> 0.001, clump window <10 kb), and utilized proxy SNPs for a very small number of missing SNPs in outcome data. Using the formula: F = beta^2^/se^2^, an F-statistic > 10 indicated a low likelihood of instrument bias (19). R^2 ^= 2 × (EAF)× (1 –EAF) × (*β*)^2^, *β* reflects the influence of SNP loci on the outcome and EAF represents the frequency of effect alleles, we use R^2^ to represent the percentage of variance in unpleasant emotions instruments (20).

### 2.4. Statistical analysis

The random effects inverse variance weighting (IVW) technique is used as the major analysis approach to investigate the association between unpleasant emotions and coronary atherosclerosis, it incorporates Wald ratio estimation to provide a consistent assessment of the causal effect of exposure on outcomes (21). Considering that IVW estimates might be affected by defective instrument bias or pleiotropy, we investigated the validity and robustness of the results by conducting sensitivity analyses using the Weighted median and MR-Egger. On the assumption that more than fifty percent of genetic variation is a useful tool variable, the weighted median technique can be used to quantify the effect (22). MR-Egger may be employed for pleiotropic estimation when the instrumental variable is invalid and there is inadequate evidence of directional pleiotropy indicated by an intercept of zero (23). Additionally, we use the Cochran Q statistic to evaluate the presence of heterogeneity (i.e., potential horizontal pleiotropy) (24) and utilize MR-Pleiotropy Residual Sum and Outlier methods (MR-PRESSO) to identify and remove probable outliers as well as reevaluate heterogeneity (25). R's TwoSampleMR (version 0.5.6) and MRPRESSO (version 1.0) tools do the analysis (version 4.2.1). *P *< 0.05 was regarded as statistically significant in the MR analysis statistical test.

## 3. Results

According to the predetermined criterion, we chose 44 SNPs that were highly related to unpleasant emotions; one of these SNPs was unavailable in the result and was substituted by an acceptable proxy SNP. Coordination of exposure and outcome data led to the elimination of palindrome sequences with low allele frequency and incompatible SNPs. Finally, 40 SNPs were chosen as instrumental factors for the investigation of the link between unpleasant emotions and coronary atherosclerosis (Supplementary Table S1). The estimated F statistics were larger than 10, suggesting that the variables were significant instrument variables (Supplementary Table S1). There was no sample repeat in the populations of exposure and outcome.

**Figure 2:**
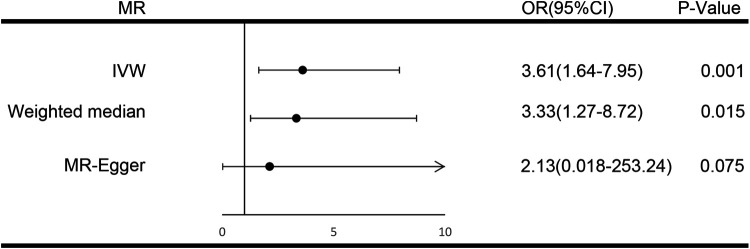
Forest plot of three Mendelian random estimators of the effect of unpleasant emotions on coronary atherosclerosis. IVW, indicates inverse variance weighted; MR, Mendelian randomization; OR, odds ratio.

**Figure 3:**
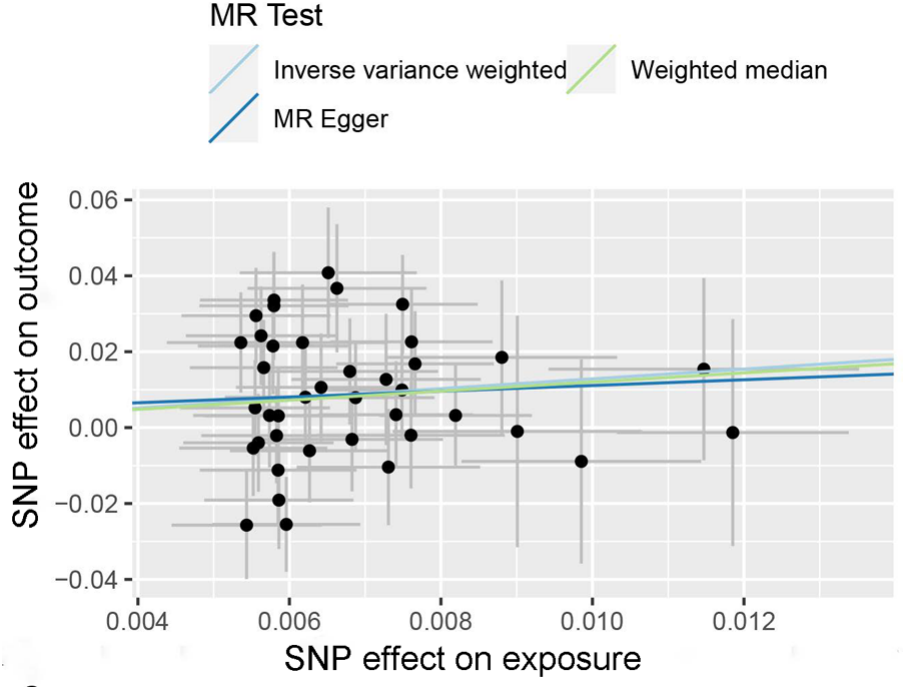
Scatterplot of Mendelian randomization estimates of genetic risk of unpleasant emotions on coronary atherosclerosis. Each point represents the SNP effects on unpleasant emotions and coronary atherosclerosis. The line at each point reflects the 95% confidence interval, the horizontal coordinate is the effect of SNP on exposure (unpleasant emotions) and the vertical coordinate is the effect of SNP on the outcome (coronary atherosclerosis). SNPs, single nucleotide polymorphisms.

**Figure 4:**
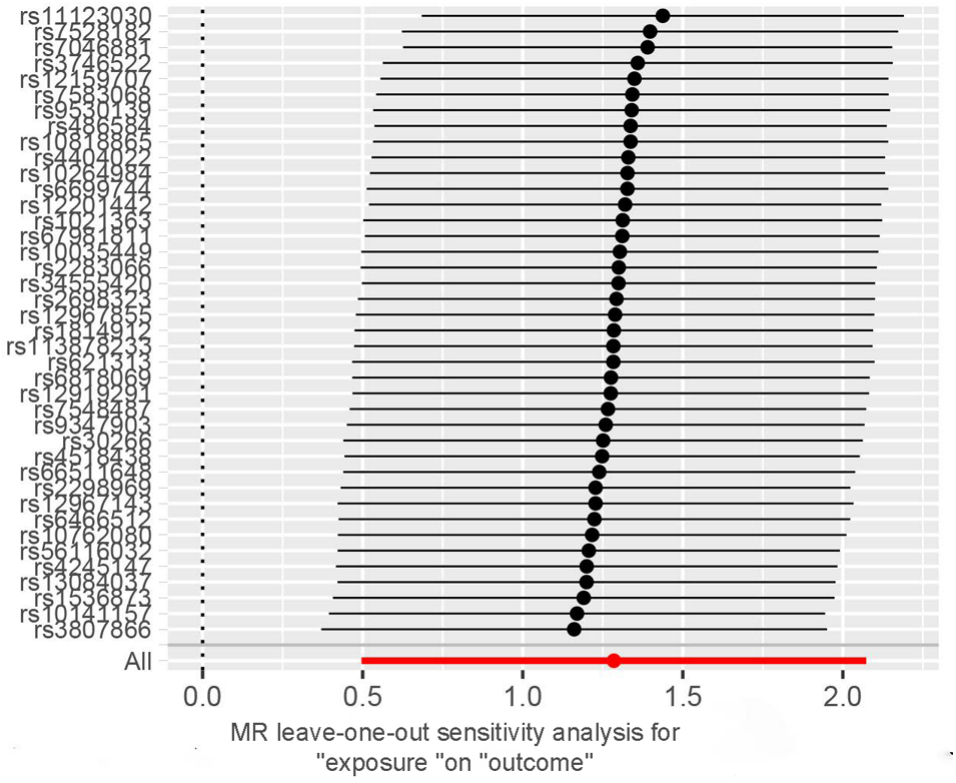
Leave-one-out analysis of unpleasant emotions on coronary atherosclerosis. Circles indicate MR estimates for unpleasant emotions and coronary atherosclerosis using the inverse-variance weighted fixed-effect method if the SNP was omitted. The bars indicate the CI of MR estimates. CI, confidence interval; MR, Mendelian randomization.

**Figure 5:**
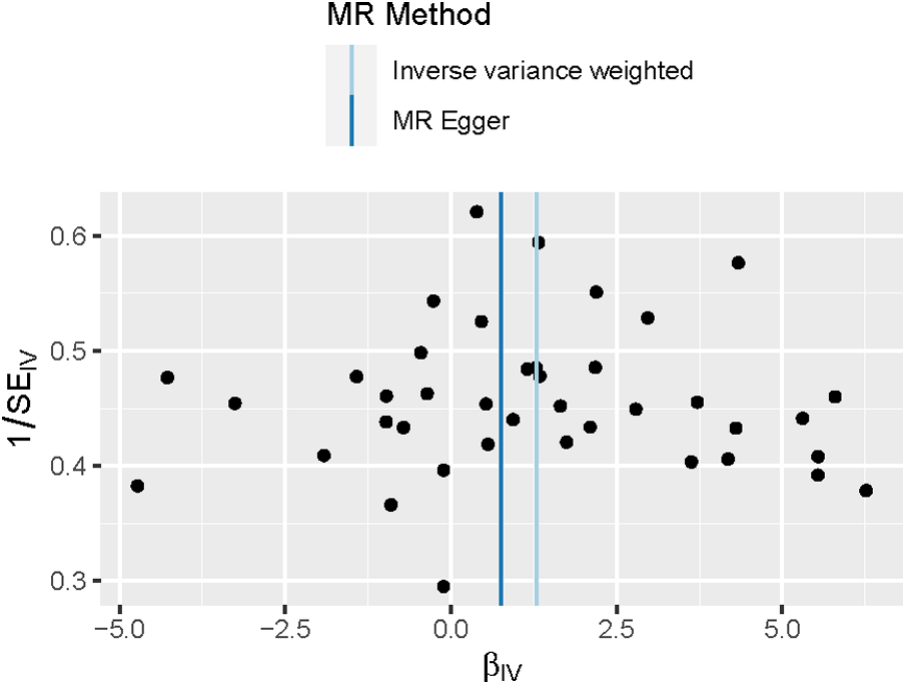
Funnel plot of the Mendelian randomization analysis for coronary atherosclerosis. The funnel plot assessed the existence of probable heterogeneity among the estimations, which suggests the possibility of pleiotropic effects. The graph depicts the observed causal impact of each of the 40 instrumental variables (IVs) as dots, and the average causal effect of all IVs combined (bIV) using the inverse variance weighted and MR-Egger approach on the x-axis. The Y axis indicates the inverse standard error of the predicted causal impact for each single nucleotide polymorphism (SNP).

For each unit increase in the genetically determined log-transformed likelihood of unpleasant feelings, the odds ratio for coronary atherosclerosis increased by 3.61 (95% CI: 1.64–7.95; *P *= 0.001). Unpleasant emotions are strongly associated with the risk of coronary atherosclerosis, suggesting a hereditary predisposition. Weighted median [OR, 3.33; 95%CI, 1.27–8.72, *P *= 0.015] and MR Egger [OR, 2.13; 95%CI, 0.018–253.24, *P *= 0.75] produced equivalent results, although with larger confidence intervals for MR Egger, yet in the same direction (Figure 2). The scatter plot illustrates the association between the SNP effect on unpleasant emotions and the SNP effect on coronary atherosclerosis (Figure 3). A leave-one-out analysis (Figure 4) and a single SNP analysis (Supplementary Figure S1) indicated that no single SNP was responsible for the observed connection. There was minimal variability in the causative estimates of 40 SNPs. It did not detect heterogeneity [Cochran Q = 53.68(*P *= 0.059)] and the MR-Egger(*P *= 0.828) or MR- PRESSO global tests (*P *= 0.067) could not discover any pleiotropic effects. MR- PRESSO did not uncover any abnormal SNPs yet. The funnel plot depicts the aforementioned findings (Figure 5).

## 4. Discussion

This research revealed convincing evidence of a causal relationship between genetically predicted unpleasant emotions and the probability of developing coronary atherosclerosis.

Coronary atherosclerosis, also known as coronary artery disease (CAD), is the most prevalent type of cardiovascular disease (CVD) (1). Many observational studies support the assumption that anxiety or depression is related to an increased risk of CVD, thereby corroborating the results of our MR investigations. In a cross-sectional study, the researchers used Beck Anxiety Inventory (BAI) (26) and Beck Depression Inventory (BDI) (27) to assess the severity of anxiety or depressive tendencies in participants who had never been diagnosed with a psychiatric disorder (28). More than 58% of the coronary slow flow (CSF) group had a BAI score of more than 22 points, and there was a substantial association between the BAI score and the severity of CSF and the number of injured vessels, and each unit increase in the BDI was associated with a 1.2% increase in the probability of CSF occurrence (*P* = 0.001) (28). This is also supported by a recent meta-analysis of depressed symptoms with subclinical atherosclerosis and the National Epidemiologic Survey on Alcohol and Related Conditions (NESARC) (29, 30). It is indicated that despondent mood, loss of interest or delight, and fatigue were more likely to be linked to subclinical atherosclerosis (29). And the risk of developing CAD is 2.01 to 2.09 times higher than normal after one year of persistent major depressive disorder (MDD) and/or generalized anxiety disorder (GAD) (30). However, there is contradictory evidence about the effect of unpleasant emotions on atherosclerosis (31–34). In a long-term observational study where children's atherosclerosis was measured using pulse wave velocity (PWV), logistic regression showed that high brachial-ankle PWV (baPWV) was independently associated with depressive and anxious symptoms, such as feeling sick in the bath, headaches, general fatigue, anxiety, and emotional ups and downs, while low baPWV was associated with motivation and good friendships (31). Another meta-analysis of 15 studies on the association between optimism and all-cause mortality at risk of future cardiovascular events in 229,391 individuals revealed that optimism was substantially related to a lower risk of cardiovascular events (*RR *= 0.65, *95%CI*: 0.51–0.78, *P *< 0.001) (33). In contrast, there was no relationship between the Center for Epidemiological Studies-Depression (CES-D) (35) scale scores and carotid intima-media thickness (cIMT) (36), an alternative marker of subclinical atherosclerosis in participants, in the Baltimore longitudinal study of aging (*P *= 0.68) (32). In the Dallas Heart Study, after adjusting for confounding variables such as age, gender, and race, there was no correlation between the Quick Inventory of Depressive Symptomatology-Self Report (QIDS) score (37), which is a standardized instrument used to measure the severity of depressive symptom in terms of difficulty sleeping, waking easily, feeling sad, appetite and weight changes, reduced concentration, inability to accept oneself, suicidal thoughts, reduced interest, and energy, slowed thinking, fidgeting, etc. in the previous week, and the occurrence or severity of CAC (*β*=0.088, *P* = 0.240, *OR* = 1.092, *95% CI*: 0.943–1.264), a marker of coronary atherosclerosis (34, 38). Multiple plausible explanations exist for this contradictory conclusion. Firstly, the information collected from psychosocial measures, such as the CES-D, could differ based on the socio-cultural environment of different geographic regions and countries, as the samples are from different periods and locations (32). Secondly, plaque and IMT may have distinct associations with CES-D or BAI scores; since some studies have examined IMT without considering carotid plaque, there may be no association between the two (32). Thirdly, in certain cross-sectional observational studies, unpleasant symptoms and atherosclerosis examined at a particular time point may not be indicative of changes over time and herein resides one of the limitations of observational research (34). Finally, the mechanism by which unpleasant symptoms influence atherosclerosis may not involve calcification, and there may be other indicators besides CAC that are affected (34). Consistent with earlier MR research on the risk of cardiovascular illness connected with psychological variables, our findings provide solid estimates of causality (39–41). However, they primarily focused on depression and disease symptoms and did not investigate the effect on the fundamental pathological alterations of CVD (40). Moreover, the odds ratio for determining the degree of the impact is less than our findings (1.14 vs. 3.61), suggesting that anxiety or tension may amplify this effect (40). Although the design of our analysis was comparable to that of Li et al. (41), we added some novel elements. Firstly, they selected CAD as one of the outcome variables; strictly speaking, coronary atherosclerosis is a pathological change that precedes CAD, and our study is essential to comprehending the influence of emotion on CVD. Secondly, they used self-reported broad depression as a diagnostic adjunct, which may have been influenced by other psychiatric symptoms/disorders (42). We used self-perceived unpleasant emotions as an exposure variable to include more data and thus to support the causal involvement of psychosocial factors in CAD. Anxiety and depression may potentially influence the establishment and progression of atherosclerosis.

Several pathophysiological pathways may explain the causative impact of psychological disorders, such as anxiety or depression, on the risk of atherosclerosis or coronary heart disease. Psychosocial stress may result in noncompliance, and unhealthy lifestyles, together with a higher risk of coronary artery disease due to hazardous health behaviors (43). In addition, chronic inflammation, endothelial dysfunction, and elevated platelet activity may serve as a connecting factor (44, 45).

The key advantage of this study is the design of MR, which may mimic natural randomization and reduce residual confounding to get precise causal estimates. Additionally, the GWAS meta-analysis that we used was based on the European population, whose main genetic components were used to alter the population structure. There is no overlap between samples of exposure and outcome variables, therefore we do not anticipate population stratification bias or weak instrumental variables to affect our results. Since the F statistic value is more than 10, it is evident that the instrumental variables we selected are reliable, but we must exercise care when disseminating the study results to other populations.

However, it is important to keep in mind that this study has a few limitations. Firstly, we cannot rule out the possibility that our study was affected by unknown confounding variables, but we have applied a series of sensitivity analysis methods to spot and correct likely confounding and pleiotropy. Secondly, since we employ summary-level data, individual-level information, including biochemical signs, hinders our investigation of the underlying mechanisms of connection. Thirdly, it is difficult to evaluate the relative impact of anxiety or depression on the development or progression of coronary atherosclerosis. Nonetheless, this study properly explains the influence of psychosocial factors on coronary atherosclerosis.

## 5. Conclusion

This research supports the causative significance of anxiety and depression, among other psychosocial variables, in coronary atherosclerosis. It is imperative that we treat psychosocial stress as a modifiable risk factor for all of our patients and apply stress management approaches to provide patients with extra advantages.

## Data Availability

Publicly available datasets were analyzed in this study. This data can be found here: https://gwas.mrcieu.ac.uk/.
