# Supplementary material for: Using Mendelian randomization analysis to determine the causal connection between unpleasant emotions and coronary atherosclerosis
Source: Front Cardiovasc Med. 2023 May 22;10:1126157. doi: 10.3389/fcvm.2023.1126157 (PMC10239874; doi:10.3389/fcvm.2023.1126157)

**Figure S1. Forest plot of single SNP analysis.**

Each horizontal solid line reflects the results of individual SNPs estimated using the Wald ratio method


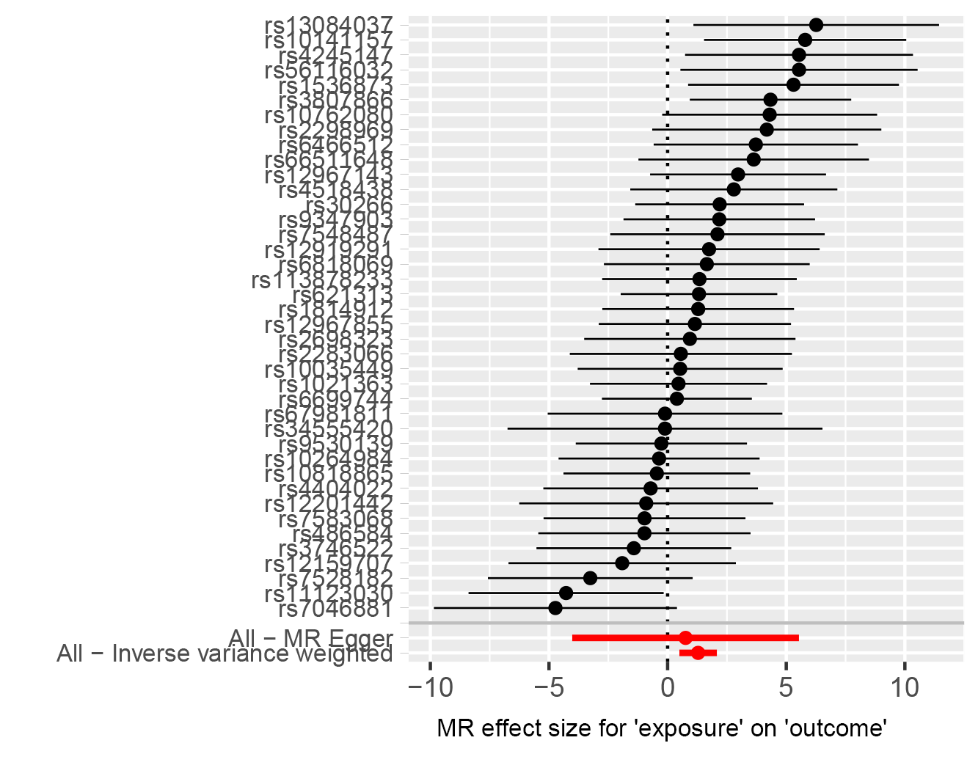

Supplement: Supplementary file 1 [file Datasheet1.docx]
