# Supplementary material for: Using Mendelian randomization analysis to determine the causal connection between unpleasant emotions and coronary atherosclerosis
Source: Front Cardiovasc Med. 2023 May 22;10:1126157. doi: 10.3389/fcvm.2023.1126157 (PMC10239874; doi:10.3389/fcvm.2023.1126157)
Supplement: Supplementary file 2 [file Table1.docx]

| **Table S1. List of 40 single nucleotide polymorphisms(SNPs) used as genetic instruments for unpleasant emotions and their associations with coronary atherosclerosis** | | | | | | | | | | | | | | | | | |  |
| --- | --- | --- | --- | --- | --- | --- | --- | --- | --- | --- | --- | --- | --- | --- | --- | --- | --- | --- |
| **SNP** | **Effect allele** | **Other allele** | **Effect allele frequency** | **unpleasant emotions** | | |  | | **coronary atherosclerosis** | | | | | | **R^2^** | | **F** | |
|  |  |  |  | **Beta** | **SE** | ***P* value** |  |  | **Beta** | | **SE** | | ***P* value** | |  |  |  |  |
| rs10035449 | C | T | 0.533252 | 0.005854 | 0.000977481 | 2.10E-09 | | 0.0031 | | 0.0129 | | 0.8096 | | 1.70596E-05 | | 35.86807555 | |  |
| rs10141157 | C | T | 0.513815 | 0.005797 | 0.00097716 | 3.00E-09 | | 0.0336 | | 0.0126 | | 0.00788007 | | 1.67873E-05 | | 35.18943602 | |  |
| rs1021363 | G | A | 0.643587 | -0.00741 | 0.00102059 | 3.90E-13 | | -0.0034 | | 0.0141 | | 0.8103 | | 2.5172E-05 | | 52.67739458 | |  |
| rs10264984 | T | C | 0.409418 | 0.005831 | 0.000994135 | 4.50E-09 | | -0.0021 | | 0.0126 | | 0.8678 | | 1.644E-05 | | 34.39796807 | |  |
| rs10762080 | G | A | 0.584876 | -0.00563 | 0.000992175 | 1.40E-08 | | -0.0242 | | 0.013 | | 0.0625302 | | 1.53709E-05 | | 32.15527508 | |  |
| rs10818865 | G | A | 0.209045 | 0.006826 | 0.00120174 | 1.30E-08 | | -0.0031 | | 0.0137 | | 0.8188 | | 1.54097E-05 | | 32.26643465 | |  |
| rs11123030 | C | T | 0.510908 | -0.00596 | 0.000977052 | 1.10E-09 | | 0.0255 | | 0.0125 | | 0.0422503 | | 1.77466E-05 | | 37.19779915 | |  |
| rs113878233 | T | C | 0.061019 | 0.01147 | 0.00204987 | 2.20E-08 | | 0.0154 | | 0.024 | | 0.5208 | | 1.50765E-05 | | 31.31099829 | |  |
| rs12159707 | A | G | 0.344175 | -0.00585 | 0.001034 | 1.50E-08 | | 0.0112 | | 0.0143 | | 0.4309 | | 1.54507E-05 | | 32.01173829 | |  |
| rs12201442 | G | A | 0.106529 | 0.009853 | 0.00158252 | 4.80E-10 | | -0.0089 | | 0.0269 | | 0.741401 | | 1.84808E-05 | | 38.76536166 | |  |
| rs12919291 | C | G | 0.18922 | 0.007276 | 0.00124931 | 5.70E-09 | | 0.0127 | | 0.0173 | | 0.4624 | | 1.62451E-05 | | 33.92194617 | |  |
| rs12967143 | C | G | 0.699435 | -0.00761 | 0.00107088 | 1.20E-12 | | -0.0226 | | 0.0144 | | 0.1177 | | 2.43626E-05 | | 50.52731418 | |  |
| rs12967855 | G | A | 0.6688 | -0.00687 | 0.00104168 | 4.20E-11 | | -0.0079 | | 0.0142 | | 0.5761 | | 2.09302E-05 | | 43.54000613 | |  |
| rs13084037 | A | G | 0.774396 | -0.00651 | 0.0011683 | 2.50E-08 | | -0.0408 | | 0.0172 | | 0.01799 | | 1.48211E-05 | | 31.07640202 | |  |
| rs1536873 | A | G | 0.468978 | 0.005559 | 0.000984187 | 1.60E-08 | | 0.0295 | | 0.0126 | | 0.01894 | | 1.53938E-05 | | 31.90773011 | |  |
| rs1814912 | C | T | 0.325616 | 0.006211 | 0.00106149 | 4.90E-09 | | 0.008 | | 0.0128 | | 0.5331 | | 1.6941E-05 | | 34.23444364 | |  |
| rs2283066 | C | T | 0.3755 | -0.00574 | 0.00100863 | 1.30E-08 | | -0.0032 | | 0.0137 | | 0.8168 | | 1.5438E-05 | | 32.35607981 | |  |
| rs2298969 | G | A | 0.481488 | -0.00536 | 0.000977753 | 4.20E-08 | | -0.0224 | | 0.0132 | | 0.0889099 | | 1.43399E-05 | | 30.04097765 | |  |
| rs2698323 | C | T | 0.415706 | 0.005547 | 0.00098974 | 2.10E-08 | | 0.0052 | | 0.0126 | | 0.6818 | | 1.49454E-05 | | 31.40625464 | |  |
| rs30266 | A | G | 0.32822 | 0.007659 | 0.00104038 | 1.80E-13 | | 0.0168 | | 0.0139 | | 0.226 | | 2.58649E-05 | | 54.18818164 | |  |
| rs34555420 | T | G | 0.097832 | -0.00901 | 0.00164304 | 4.20E-08 | | 0.001 | | 0.0305 | | 0.9731 | | 1.43248E-05 | | 30.06043037 | |  |
| rs3746522 | T | C | 0.215906 | 0.007307 | 0.00121043 | 1.60E-09 | | -0.0104 | | 0.0153 | | 0.4964 | | 1.80762E-05 | | 36.43882661 | |  |
| rs3807866 | A | G | 0.410746 | 0.007494 | 0.000990903 | 3.90E-14 | | 0.0325 | | 0.013 | | 0.0124899 | | 2.71845E-05 | | 57.19439896 | |  |
| rs4245147 | T | C | 0.514502 | 0.005796 | 0.000984572 | 3.90E-09 | | 0.0321 | | 0.0142 | | 0.0241201 | | 1.67801E-05 | | 34.64929099 | |  |
| rs4404022 | T | A | 0.416532 | 0.005591 | 0.000992331 | 1.80E-08 | | -0.004 | | 0.0129 | | 0.756 | | 1.51951E-05 | | 31.74646579 | |  |
| rs4518438 | C | T | 0.510117 | -0.00566 | 0.000975737 | 6.60E-09 | | -0.0158 | | 0.0126 | | 0.2097 | | 1.60176E-05 | | 33.66194252 | |  |
| rs486584 | T | C | 0.523787 | 0.005523 | 0.000977235 | 1.60E-08 | | -0.0054 | | 0.0126 | | 0.668199 | | 1.5216E-05 | | 31.93860095 | |  |
| rs56116032 | G | A | 0.219452 | -0.00663 | 0.00118115 | 2.00E-08 | | -0.0367 | | 0.0169 | | 0.0295897 | | 1.50454E-05 | | 31.47930557 | |  |
| rs621313 | G | A | 0.49123 | 0.007486 | 0.000979968 | 2.20E-14 | | 0.0099 | | 0.0126 | | 0.4351 | | 2.8008E-05 | | 58.34737492 | |  |
| rs6466512 | A | G | 0.417265 | -0.00578 | 0.000992323 | 5.60E-09 | | -0.0215 | | 0.0127 | | 0.0917107 | | 1.62662E-05 | | 33.96771662 | |  |
| rs66511648 | C | T | 0.284735 | 0.006173 | 0.00108633 | 1.30E-08 | | 0.0224 | | 0.0153 | | 0.1437 | | 1.55232E-05 | | 32.29394252 | |  |
| rs6699744 | T | A | 0.615966 | 0.008193 | 0.00100769 | 4.30E-16 | | 0.0032 | | 0.0132 | | 0.8069 | | 3.17561E-05 | | 66.10239117 | |  |
| rs67981811 | G | C | 0.11416 | -0.01185 | 0.00153202 | 1.00E-14 | | 0.0013 | | 0.0299 | | 0.9649 | | 2.84107E-05 | | 59.8486579 | |  |
| rs6818069 | G | T | 0.255475 | 0.006418 | 0.001125 | 1.20E-08 | | 0.0106 | | 0.0142 | | 0.4565 | | 1.56705E-05 | | 32.54758282 | |  |
| rs7046881 | G | T | 0.423242 | -0.00543 | 0.000990133 | 4.00E-08 | | 0.0257 | | 0.0142 | | 0.0700697 | | 1.44202E-05 | | 30.12812273 | |  |
| rs7528182 | T | C | 0.433177 | 0.005861 | 0.00098508 | 2.70E-09 | | -0.0191 | | 0.0129 | | 0.1401 | | 1.68681E-05 | | 35.39819957 | |  |
| rs7548487 | G | A | 0.116216 | 0.008804 | 0.0015217 | 7.20E-09 | | 0.0185 | | 0.0203 | | 0.361 | | 1.59225E-05 | | 33.4742508 | |  |
| rs7583068 | A | T | 0.319601 | 0.006266 | 0.00104958 | 2.40E-09 | | -0.0061 | | 0.0136 | | 0.6541 | | 1.70744E-05 | | 35.63802649 | |  |
| rs9347903 | T | C | 0.226314 | 0.006798 | 0.00116505 | 5.40E-09 | | 0.0148 | | 0.014 | | 0.2894 | | 1.61824E-05 | | 34.04445292 | |  |
| rs9530139 | T | C | 0.194414 | -0.0076 | 0.00123719 | 7.90E-10 | | 0.002 | | 0.014 | | 0.887 | | 1.8115E-05 | | 37.78305592 | |  |
